# Characterization of feeding skills and clinical markers of preterm newborns with gastroschisis in a neonatal therapy unit

**DOI:** 10.1590/2317-1782/20232022145en

**Published:** 2023-08-21

**Authors:** Fernanda Chiarion Sassi, Ana Paula Ritto, Daniela Chiarion Sassi, Ana Carla Ansuino, Gisele Chagas de Medeiros, Fabiola Juste, Débora Maria Béfi-Lopes, Claudia Regina Furquim de Andrade

**Affiliations:** 1 Departamento de Fisioterapia, Fonoaudiologia e Terapia Ocupacional, Faculdade de Medicina, Universidade de São Paulo - USP - São Paulo (SP), Brasil; 2 Hospital das Clínicas, Faculdade de Medicina, Universidade de São Paulo - USP - São Paulo (SP), Brasil; 3 Hospital Regional de Cotia - Cotia (SP), Brasil

**Keywords:** Speech Language and Hearing Sciences, Newborn, Infant Premature, Gastroschisis, Alternative Feeding, Fonoaudiologia, Recém-Nascido, Recém-Nascido Prematuro, Gastrosquise, Alimentação Alternativa

## Abstract

**Objective:**

To describe the clinical and feeding findings of premature infants with gastroschisis (GTQ) in a neonatal intensive care unit and compare them to preterm infants (NBs) without congenital anomalies.

**Methods:**

A retrospective case-control study was conducted with 50 premature NBs (25 with GTQ and 25 without comorbidities - control group). The NBs were compared regarding demographic and clinical data: risk of mortality and speech-language assessment (nonnutritive and nutritive sucking). Subsequently, a multiple logistic regression model was applied to determine the variables associated with the negative speech therapy outcome (speech therapy discharge after more than 7 days considering the first speech therapy evaluation).

**Results:**

The results of the first analysis indicated that there was a difference between the GTQ and the CG for the following variables: total time in days of hospitalization; use of mechanical ventilation (invasive x noninvasive); days of life on the date of the first speech-language assessment; use of alternative feeding route; and the GTQ group had worse results. The results of the multiple logistic regression indicated that the diagnosis of GTQ, the use of invasive mechanical ventilation, and the absence of adequate intraoral pressure during the first speech-language evaluation were risk factors for a negative speech-language outcome.

**Conclusion:**

The feeding skills of preterm infants with QTG are related to the severity of the condition (gastrointestinal tract complications), requiring longer hospitalization, use of invasive mechanical ventilation, prolonged use of alternative feeding route and requiring more speech therapy to start oral feeding.

## RESUMO

**Objetivo:**

Descrever os achados clínicos e de alimentação de prematuros com gastrosquise (GTQ) em uma unidade de terapia intensiva neonatal comparando-os a recém-nascidos (RNs) prematuros sem anomalias congênitas.

**Método:**

Foi realizado estudo caso controle de caráter retrospectivo com 50 RNs prematuros (25 com GTQ e 25 sem comorbidades - grupo controle). Os RNs foram comparados quanto aos dados demográficos clínicos: risco de mortalidade e de avaliação fonoaudiológica (sucção não nutritiva e nutritiva). Posteriormente foi aplicado o modelo de regressão logística múltipla a fim de determinar as variáveis associadas ao desfecho fonoaudiológico negativo (alta fonoaudiológica após mais de 7 dias considerando a primeira avaliação fonoaudiológica).

**Resultados:**

Os resultados da primeira análise indicaram que houve diferença entre o GTQ e o GC para as seguintes variáveis: tempo total em dias de internação; uso de ventilação mecânica (invasiva x não invasiva); dias de vida na data da primeira avaliação fonoaudiológica; uso de via alternativa de alimentação, sendo que o grupo GTQ apresentou piores resultados. Os resultados da regressão logística múltipla indicaram que o diagnóstico de GTQ, uso de ventilação mecânica invasiva e ausência de pressão intraoral adequada durante a primeira avaliação fonoaudiológica foram fatores de risco para o desfecho fonoaudiológico negativo.

**Conclusão:**

As habilidades de alimentação dos prematuros com GTQ está relacionada à gravidade do quadro (complicações do trato gastro digestivo), exigindo maior tempo de internação, uso de ventilação mecânica invasiva, uso prolongado de via alternativa de alimentação e necessidade de mais atendimento fonoaudiológico para iniciar a alimentação por via oral.

## INTRODUCTION

Gastroschisis (GTQ) is characterized by a congenital defect in the closure of the abdominal wall with herniation of the intestines and other abdominal organs to the amniotic cavity^(1-3)^. It is considered a sporadic event with multifactorial etiology and is associated with low maternal age. According to the literature, the incidence rate of GTQ varies from 1 to 4.5 per 10.000 live births per year and the mortality rate related to GTQ alone is approximately 3.6% of cases ^(4,5)^. It is considered one of the most common causes of indication for surgery in neonates and is associated with the increased need for intensive care and parenteral nutrition, mainly due to the gastrointestinal changes characteristic of this malformation ^(6,7)^. Therefore, for the best care and prognosis, an entire multidisciplinary team needs to assist these neonates, including speech therapists ^(8)^.

Among the many challenges present in the routine of the neonatal intensive care unit (NICU), the introduction and management of safe and efficient oral feeding for premature infants is an important aspect in the care of this population. However, as there is no consensus in the literature on which would be the ideal strategy to feed newborns after intestinal interventions for primary gastrointestinal diseases, a great variability of procedures are observed, as well as increased risk for the development of feeding-related complications (e.g., the presence of necrotizing enterocolitis) ^(9,10)^. Food intolerance due to paralytic ileus may hinder the identification of necrotizing enterocolitis, resulting in a delay in the feeding progression of infants who already have a very fragile condition ^(11)^.

In general, the existing protocols suggest that feeding be initiated with the provision of a minimum enteral volume associated with parenteral feeding as early as possible, especially considering surgical cases ^(11,12)^. The final clinical objective is to achieve an adequate volume in enteral feeding, with weight gain and good tolerance to oral feeding. Only after this, should attempts to withdraw from the alternative feeding route begin. The literature indicates that the supply of minimum enteral volume is a risk factor for liver disease due to intestinal failure, which may lead to the need for liver transplantation ^(11)^. The etiology of liver disease in these cases is multifactorial and is usually associated with prematurity, prolonged use of parenteral nutrition (>30-60 days), absence of enteral nutrition and presence of infections ^(11,12)^. Considering that the indicators of intestinal failure are not clear during the preoperative period, the optimization of enteral nutrition for at-risk newborns is advisable, even when considering the need for clinical stability for the initiation of oral feeding.

When considering the child who needs special care at the beginning of his life, such as those who still perform large-scale surgical interventions in the early hours post-birth to correct QCT, oral feeding can be a differential in the recovery and future quality of life, not only for the child but also for the family ^(13,14)^. Safe and efficient oral feeding avoids aspiration of food and does not compromise the respiratory state of the newborn. It is noteworthy that full oral feeding of the NB is considered one of the most important prerequisites for hospital discharge in the NICU ^(13-15)^.

The main objectives of the evaluation and speech-language intervention with newborns in the NICU include the safe determination of the ideal time for the transition from the alternative feeding route to the oral route. The clinical speech-language assessment in this context begins with an in-depth survey of the history of the NB and then proceeds to the direct handling of the oral dysfunctions, such as weak sucking, incoordination between breathing-sucking-swallowing, or even oral hypersensitivity that hinders the acceptance and transition to oral feeding ^(16)^. These dysfunctions may be caused by factors related to various pathologies, such as GTQ, which may interfere with oral feeding ^(15,16)^.

Currently, few studies describe the speech-language findings related to swallowing and oral feeding in neonates with QTG, and it is of great importance that the speech therapist knows these changes to perform the intervention efficiently and safely, facilitating the transition from the alternative route of feeding to the oral route, safely and reducing food refusal. Thus, the present study aimed to describe the clinical and feeding findings of premature infants with QTG in a neonatal intensive care unit of a high-complexity pediatric hospital, comparing them to preterm infants without congenital anomalies.

## METHODS

This is a retrospective case-control study. The project was approved by the Ethics Committee for Analysis of Research Projects of our Institution (CAPPesq Process 2.289.029). The study data were obtained based on the analysis of medical records; therefore, the participants’ relatives waived the informed consent form.

### Participants

Participants of this study were patients born preterm, diagnosed with GTQ, submitted to a feeding evaluation at the Neonatal Intensive Care Unit, upon medical request, by the Division of Oral Myology of *Hospital das Clínicas*, between January 2017 and January 2019, at the *Instituto da Criança do Hospital das Clínicas*, School of Medicine of the University of São Paulo.

The inclusion criteria adopted for the group with GTQ were a) preterm newborns (˂37 weeks of gestational age); b) present medical diagnosis at birth of GTQ; c) no neurological changes; e) no genetic syndrome; f) not gastrostomized; and g) have been submitted to a feeding assessment by a speech-language pathologist upon medical request.

For comparison, a group of healthy children born preterm, without comorbidities (control group), paired by gestational age and subjected to the same speech-language procedures as the group of children with GTQ was included. The inclusion criteria for the control group were a) preterm newborns (˂37 weeks of gestational age); b) no neurological changes; c) no genetic syndrome; d) no gastrointestinal diseases; e) no congenital malformation; f) not having been subjected to parenteral nutrition; g) not gastrostomized; and h) having been subjected to a feeding assessment by a speech-language therapist upon medical request. The patients in the control group were selected by simple randomization. For this purpose, all patients admitted to the institution who met the criteria for inclusion in this group were identified. From this population of eligible patients, the sample was randomly selected, in which each eligible patient had the same probability of being randomly selected to form the sample.

After the application of the inclusion criteria, the final study sample consisted of 50 children born preterm, with 25 preterm infants with GTQ and 25 preterm infants without comorbidities.

### Procedures

The stages of data gathering involved clinical and demographic data, predictors of neonatal mortality using the Score for Neonatal Acute Physiology - Perinatal Extension (SNAPPE II) ^(17)^ and speech-language evaluation of nonnutritive and nutritive sucking.

### Clinical Data

Based on the medical records, the following clinical data were collected: days of life at the time of the speech-language evaluation (first evaluation); route of feeding at the time of the first speech-language evaluation (exclusive orogastric tube, exclusive nasoenteral tube, exclusive oral feeding, mixed feeding); speech therapy conducted after the first evaluation (exclusive orogastric tube, exclusive nasoenteral tube, exclusive oral feeding, mixed feeding); number of speech therapy sessions for oral feeding introduction; number of speech therapy visits between the completion of the first evaluation and the removal of the alternative feeding route; time to speech therapy discharge (in days); and time to hospital discharge (in days). The number of visits was recorded in revenue value units (RVUs), i.e., each RVU represents 15 minutes of the speech therapy intervention time.

In addition, clinical data from birth were also collected to complement the data analysis, namely: gestational age; corrected gestational age; classification of preterm newborn; classification of size at birth; classification of birth weight adequacy ^(18)^; use of invasive mechanical ventilation; use of noninvasive mechanical ventilation; time of orotracheal intubation (in days) and time of orotracheal extubation until the time of evaluation. All data mentioned above were defined based on the medical criteria of the neonatal unit.

For the GTQ group, in addition to all the aforementioned data, the following clinical data were also collected: presence of serositis; presence of paralytic ileus; presence of intestinal atresia; presence of sepsis; presence of cholestasis; need for anastomosis; presence of necrotizing enterocolitis; presence of liver diseases; presence of gastrointestinal complications (e.g. , biliary lithiasis, intestinal subocclusion, obstructive abdomen); time of alternative feeding route until the first speech-language evaluation (in days); and time of parenteral nutrition (in days).

### Risk of mortality of the newborn

To determine the risk of mortality of the newborn, the Score for Neonatal Acute Physiology, Perinatal Extension - Version II (SNAPPE-II) ^(17)^ was used. The score of this scale ranges from 0 to 115 and the higher the score, the greater the risk of patient mortality.

### Clinical speech-language evaluation of nonnutritive (NNS) and nutritive (NS) sucking

Feeding abilities were assessed using a protocol adopted at the institution ^(16,19)^. This protocol includes the assessment of body organization, nonnutritive sucking, sucking rhythm, oral reflexes, evaluation of clinical signs of risk for bronchoaspiration and clinical performance with volume supply. The application of the protocol is divided in two stages: evaluation of nonnutritive sucking (absence of fluid supply, e.g. , gloved finger or pacifier) and evaluation of nutritive sucking (supply of milk from the breast or bottle). The protocol does not allow interpretation of the results obtained, but only indicates the efficiency of the functions analyzed. The record of each item of the protocol is performed between present, absent and insufficient.

It is important to recognize that effective oral feeding in newborns is based not only on adequate sucking, but also on the coordination between sucking- swallowing-breathing. In addition, regardless of such abilities, the behavioral state of the infant and its organization during feeding can significantly affect the performance of the newborn.

The criteria for data interpretation were as follows: 1) body organization (present - limbs flexed, midline; absent - limbs in extension); 2) intraoral pressure (present - full milk extraction to the mouth; absent - no milk extraction by the mouth; insufficient - little milk extraction to the mouth); sucking rhythmic movement (present - maintains sucking rhythm per pause; absent - does not maintain sucking rhythm per pause).

### Data analysis

The data collected underwent statistical analysis using SPSS software version 27. The quantitative data received descriptive analysis (mean and standard deviation) and univariate inferential analysis comparing the groups (Mann-Whitney test). The qualitative data received descriptive analysis (total count and percentage) and univariate inferential analysis comparing the groups (Pearson’s chi-square test). The level of significance adopted in all analyses was 5%.

Subsequently, the 50 participants were divided into two groups according to the results of the speech therapy treatment: participants who were discharged from speech therapy up to 7 days after the first speech-language evaluation were considered to have a positive outcome (19 participants. or 38.0% of the sample), and participants who were discharged after 7 days were considered to have a negative outcome. According to our Institution’s quality indicators, any infant who remains 7 days or more under feeding stimulation (speech therapy treatment) is considered at risk due to the exposure to situations that do not favor adequate overall development. The risk indicators for prolonged speech therapy during hospitalization were analyzed considering this parameter. The intention of this analysis was to identify which prognostic indicators were the most significant predictors of positive speech-language outcomes in the study population. Univariate analysis was initially performed to verify the strength of the association of the variables tested between the groups with the shortest and longest speech therapy treatments. A risk analysis was then performed to determine which characteristics were associated with the negative outcome (i.e., speech therapy discharge more than seven days after the initial speech-language assessment). Variables with a p value less than or equal to 0.20 in the bivariate analyses described above and with clinical plausibility were included in the analysis. The multiple logistic regression model was adjusted to jointly evaluate the association of the characteristics of interest that may influence the negative outcome and the *stepwise backward* selection method was used with input and output criteria of the variables of the final model at 5%.

## RESULTS

- Univariate intergroup analysis-case-control comparison:

The GTQ and control groups were compared according to Tables 1 and 2. The results presented in Table 1 indicate that the GTQ group and the control group (CG) showed significant differences for the following variables: total hospitalization time (GTQ> CG); number of participants who used invasive mechanical ventilation (GTQ> CG); and number of participants who used noninvasive mechanical ventilation (GTQ> CG).

**Table 1:** Comparison between the control and research groups for demographic and clinical variables and mortality risk

	**GTQ Group (n=25)**	**Control Group (n=25)**	**Total (n=50)**	**p value**
**Gestational age at birth. in weeks**				
mean (± SD)	34.4 (±1.8)	34.4 (±1.8)	34.4 (±1.8)	>0.999
median (min; max)	35.0 (28; 36)	35.0 (28; 36)	35.0 (28; 36)	
**Gender. n (%)**				
Male	11 (44.0%)	11 (44.0%)	22 (44.0%)	>0.999
Female	14 (56.0%)	14 (56.0%)	28 (56.0%)	
**Classification of preterm newborns according to gestational age**. n (%)				
Very Pre-Term (28 to 31 weeks)	1 (4.0%)	1 (4.0%)	2 (4.0%)	>0.999
Pre-Moderate Preterm (32 to 33 weeks)	5 (20.0%)	5 (20.0%)	10 (20.0%)	
Pre-Late Term (34 to 37 weeks)	19 (76.0%)	19 (76.0%)	38 (76.0%)	
**Classification of the birth weight adequacy of the preterm newborn**. n (%)				
Extremely low weight	0 (0.0%)	2 (8.0%)	2 (4.0%)	0.347
Very low weight	6 (24.0%)	5 (20.0%)	11 (22.0%)	
Low weight	19 (76.0%)	18 (72.0%)	37 (74.0%)	
**Classification of the size of preterm newborns according to gestational age**. n (%)				
Small for gestational age (SGA)	13 (52.0%)	14 (56.0%)	27 (54.0%)	0.777
Adequate for gestational age (AGA)	12 (48.0%)	11 (44.0%)	23 (46.0%)	
**SNAPPE II score**				
mean (± SD)	16.8 (±10.2)	12.6 (±8.6)	14.7 (±9.6)	0.105
median (min; max)	15.0 (0; 39)	12.0 (0; 30)	12.5 (0; 39)	
**Total length of hospital stay**. in days				
mean (± SD)	78.3 (±38.9)	39.3 (±18.8)	58.8 (±36.1)	<0.001*
median (min; max)	66.0 (28; 140)	40.0 (8; 72)	46.5 (8; 140)	
**Participants who received invasive mechanical ventilation**. n (%)	25 (100.0%)	4 (16.0%)	29 (58.0%)	<0.001**
**Time of use of invasive mechanical ventilation**. in days				
mean (± SD)	10.2 (±9.5)	6.8 (±8.3)	9.7 (±9.3)	0.647
median (min; max)	8.0 (1; 31)	3.5 (1; 19)	6.0 (1; 31)	
**Participants who received non -invasive mechanical ventilation**. n (%)	0 (0.0%)	11 (44.0%)	11 (22.0%)	<0.001**
**Time of use of noninvasive mechanical ventilation**. in days				
mean (± SD)	- -	5.0 (±5.1)	- -	- -
median (min; max)	- -	3.0 (1; 16)	- -	
**Participants who presented comorbidities and/or surgical complications of gastroschisis**. n (%)	19 (38.0%)	- -	- -	- -
**Comorbidities and/or complications of gastroschisis presented**. n (%)				
Serositis	0 (0.0%)	- -	- -	- -
Paralytic ileum	0 (0.0%)	- -	- -	- -
Intestinal atresia	5 (20.0%)	- -	- -	- -
Anastomosis	3 (12.0%)	- -	- -	- -
Sepsis	17 (68.0%)	- -	- -	- -
Cholestasis	9 (36.0%)	- -	- -	- -
Necrotizing enterocolitis	2 (8.0%)	- -	- -	- -
Liver diseases	0 (0.0%)	- -	- -	- -
Gastrointestinal complications	4 (16.0%)	- -	- -	- -
**Time of use of peripheral parenteral nutrition (PPN)**. in days				
mean (± SD)	60.4 (±37.6)	- -	- -	- -
median (min; max)	43.0 (22; 164)	- -	- -	
**Time between birth and introduction of enteral diet**. in days				
mean (± SD)	34.9 (±15.4)	- -	- -	- -
median (min; max)	33.0 (16; 67)	- -	- -	

* Significant difference according to the Mann-Whitney test;

** Significant difference according to Pearson’s chi-square test

**Caption:** n = number of participants; SD = standard deviation; min: minimum; max = maximum; SNAPPE II = *Score for Neonatal Acute Physiology. Perinatal Extension. Version II*

**Table 2:** Comparison between the Control and Research Groups for speech-language and feeding variables (n = 50)

	**GTQ Group (n=25)**	**Control Group (n=25)**	**Total (n=50)**	**p value**
**Days of life in the initial speech -language assessment**				
mean (± SD)	38.6 (±18.0)	27.1 (±13.0)	32.8 (±16.6)	0.030*
median (min; max)	33.0 (17; 78)	28.0 (5; 57)	30.0 (5; 78)	
**Corrected gestational age at the initial speech -language assessment**. in weeks				
mean (± SD)	37.6 (±2.0)	37.6 (±2.0)	37.6 (±2.0)	>0.999
median (min; max)	38.0 (32; 40)	38.0 (32; 40)	38.0 (32; 40)	
**Feeding route prior to the initial speech -language assessment**. n (%)				
Exclusive orogastric tube	7 (28.0%)	12 (48.0%)	19 (38.0%)	0.006**
Exclusive nasogastric tube	3 (12.0%)	0 (0.0%)	3 (6.0%)	
Exclusive peripheral parenteral nutrition	8 (32.0%)	0 (0.0%)	8 (16.0%)	
Exclusive oral route	3 (12.0%)	7 (28.0%)	10 (20.0%)	
Mixed (SOG or SNG or NPP + VO)	4 (16.0%)	6 (24.0%)	10 (20.0%)	
**Presence of signs during suction in the initial speech -language evaluation**. n (%)				
Body organization	23 (92.0%)	23 (92.0%)	46 (92.0%)	>0.999
Intraoral pressure	17 (68.0%)	18 (72.0%)	35 (70.0%)	0.758
Suction rhythmic movement	18 (72.0%)	21 (84.0%)	39 (78.0%)	0.306
Respiratory discomfort	3 (12.0%)	2 (8.0%)	5 (10.0%)	0.637
**Speech therapy recommendation of the feeding route after the initial speech -language evaluation**. n (%)				
Exclusive orogastric tube	5 (20.0%)	4 (16.0%)	9 (18.0%)	0.606
Exclusive nasogastric tube	1 (4.0%)	0 (0.0%)	1 (2.0%)	
Exclusive oral route	5 (20.0%)	8 (32.0%)	13 (26.0%)	
Mixed (SOG or SNG + VO)	14 (56.0%)	13 (52.0%)	27 (54.0%)	
**Time between removal of invasive mechanical ventilation and initial speech -language assessment**. in days				
mean (± SD)	24.8 (±17.3)	28.8 (±7.0)	25.4 (±16.2)	0.341
median (min; max)	20.0 (3; 63)	29.0 (20; 37)	22.0 (3; 63)	
**Time between removal of noninvasive mechanical ventilation and initial speech -language assessment**. in days				
mean (± SD)	- -	24.3 (±15.7)	- -	- -
median (min; max)	- -	21.0 (6; 56)	- -	
**Time between the introduction of enteral diet and the initial speech -language evaluation**. in days				
mean (± SD)	4.2 (±7.5)	- -	- -	- -
median (min; max)	2.0 (0; 37)	- -	- -	
**Speech therapy sessions between the initial evaluation and the introduction of oral route** in URV				
mean (± SD)	1.1 (±2.4)	0.6 (±1.1)	0.9 (±1.9)	0.640
median (min; max)	0.0 (0; 10)	0.0 (0; 4)	0.0 (0; 10)	
**Speech therapy sessions between the initial evaluation and the removal of the probe (SOG or SNG)**. in URV				
mean (± SD)	8.8 (±7.3)	6.8 (±4.2)	7.8 (±5.9)	0.563
median (min; max)	8.0 (2; 29)	6.0 (1; 18)	6.5 (1; 29)	
**Time between initial speech -language assessment and discharge from speech therapy**. in days				
mean (± SD)	22.6 (±22.2)	7.8 (±5.7)	15.2 (±17.7)	0.003*
median (min; max)	14.0 (2; 75)	7.0 (1; 23)	9.0 (1; 75)	
**Time between initial speech -language assessment and hospital discharge**. in days				
mean (± SD)	38.5 (±29.3)	11.4 (±7.0)	25.0 (±25.1)	<0.001*
median (min; max)	34.0 (2; 95)	10.0 (2; 25)	14.0 (2; 95)	

* Significant difference according to the Mann-Whitney test;

** Significant difference according to Pearson’s chi-square test

**Caption**: n = number of participants; SD = standard deviation; min: minimum; max: maximum; URV = unit of income value; SOG = orogastric tube; NGS = nasogastric tube; NPP = peripheral parenteral nutrition; VO = oral route

Regarding the speech-language and dietary variables, the results presented in Table 2 indicated significant differences relevant to the clinical condition of GTQ. The GTQ group had a higher number of days of life in the speech-language evaluation, i.e., it took longer to reach the clinical stability required for speech-language intervention; presented a higher number of participants using alternative feeding routes; and took longer to receive both speech therapy and hospital discharge.

For the analysis of the speech-language outcome, patients with GTQ and CG were considered as a single group and divided into two groups: positive speech-language outcome (discharge from speech therapy within 7 days after the initial evaluation) and negative speech-language outcome (discharge from speech-language therapy more than seven days after the initial evaluation). In this new division, 19 patients had a positive speech-language outcome (5 with GTQ and 14 healthy children born preterm) and 31 patients had a negative speech-language outcome (20 with GTQ and 11 healthy children born preterm). According to the comparative analysis presented in Table 3, it is possible to observe significant differences between the groups for the following variables: number of participants with GTQ; total length of hospital stay; number of participants who received invasive mechanical ventilation; time of use of peripheral parenteral nutrition; time between birth and introduction of enteral diet; number of participants using alternative feeding in the first speech-language assessment; number of participants with inadequate intraoral pressure in the first speech-language assessment; recommendation of alternative feeding route after the first speech-language assessment; number of speech therapy sessions to remove the alternative feeding route; time between the first speech-language assessment and discharge from speech-language therapy; and time between the first speech-language assessment and hospital discharge. For all these variables, the performance of the group with a negative outcome was worse.

**Table 3:** Univariate comparison with the speech-language outcome for demographic clinical mortality risk, speech-language and feeding variables (n = 50)

	**Positive outcome**	**Negative outcome**	**p value**
**Gestational age at birth.** in weeks			
mean (± SD)	34.8 (±1.4)	34.1 (±2.0)	0.199
median (min; max)	36.0 (32; 36)	34.0 (28; 36)	
**Gender**. n (%)			
Male	7 (36.8%)	15 (48.4%)	0.425
Female	12 (63.2%)	16 (51.6%)	
**Participants with GTQ**. n (%)	5 (26.3%)	20 (64.5%)	*0.009* **
**Classification of preterm newborns according to gestational age**. n (%)			
Very Pre-Term (28 to 31 weeks)	0 (0.0%)	2 (6.5%)	0.395
Pre-Moderate Preterm (32 to 33 weeks)	5 (26.3%)	5 (16.1%)	
Pre-Late Term (34 to 37 weeks)	14 (73.7%)	24 (77.4%)	
**Classification of the birth weight adequacy of the preterm newborn**. n (%)			
Extremely low weight	1 (5.3%)	1 (3.2%)	
Very low weight	3 (15.8%)	8 (25.8%)	0.684
Low weight	15 (78.9%)	22 (71.0%)	
**Classification of the size of preterm newborns according to gestational age**. n (%)			
Small for gestational age (SGA)	13 (68.4%)	14 (45.2%)	0.109
Adequate for gestational age (AGA)	6 (31.6%)	17 (54.8%)	
**SNAPPE II score**			
mean (± SD)	16.0 (±9.0)	14.0 (±10.0)	0.450
median (min; max)	13.0 (0; 32)	12.0 (0; 39)	
**Total length of hospital stay**. in days			
mean (± SD)	44.1 (±30.8)	67.8 (±36.6)	*0.014* *
median (min; max)	34.0 (8; 140)	60.0 (18; 137)	
**Participants who received invasive mechanical ventilation**. n (%)	7 (36.8%)	22 (71.0%)	0.018**
**Time of use of invasive mechanical ventilation**. in days			
mean (± SD)	9.3 (±11.6)	9.8 (±8.8)	0.709
median (min; max)	4.0 (1; 31)	7.0 (1; 30)	
**Participants who received non -invasive mechanical ventilation**. n (%)	2 (10.5%)	9 (29.0%)	0.125
**Time of use of noninvasive mechanical ventilation**. in days			
mean (± SD)	8.5 (±10.6)	4.2 (±3.8)	0.727
median (min; max)	8.5 (1; 16)	3.0 (1; 10)	
**Participants who presented comorbidities and/or surgical complications of gastroschisis**. n (%)	4 (21.1%)	15 (48.4%)	0.053
**Comorbidities and/or complications presented**. n (%)			
Intestinal atresia	0 (0.0%)	5 (16.1%)	0.065
Anastomosis	1 (5.3%)	2 (6.5%)	0.864
Sepsis	4 (21.1%)	13 (41.9%)	0.130
Cholestasis	4 (21.1%)	5 (16.1%)	0.660
Necrotizing enterocolitis	1 (5.3%)	1 (3.2%)	0.721
Gastrointestinal complications	1 (5.3%)	3 (9.7%)	0.577
**Time of use of peripheral parenteral nutrition (PPN)**. in days			
mean (± SD)	15.0 (±28.8)	39.6 (±43.8)	0.019*
median (min; max)	0.0 (0; 93)	34.0 (0; 164)	
**Time between birth and introduction of enteral diet**. in days			
mean (± SD)	9.1 (±18.2)	22.6 (±20.7)	0.013*
median (min; max)	0.0 (0; 67)	19.0 (0; 67)	
**Days of life in the initial speech -language assessment**			
mean (± SD)	28.2 (±14.8)	35.7 (±17.2)	0.116
median (min; max)	26.0 (5; 72)	33.0 (10; 78)	
**Corrected gestational age at the initial speech -language assessment**. in weeks			
mean (± SD)	37.6 (±1.9)	37.5 (±2.1)	>0.999
median (min; max)	38.0 (35; 40)	38.0 (32; 40)	
**Feeding route prior to the initial speech -language assessment**. n (%)			
Exclusive orogastric tube	6 (31.6%)	13 (41.9%)	0.035**
Exclusive nasogastric tube	1 (5.3%)	2 (6.5%)	
Exclusive peripheral parenteral nutrition	1 (5.3%)	7 (22.6%)	
Exclusive oral route	8 (42.1%)	2 (6.5%)	
Mixed (SOG or SNG or NPP + VO)	3 (15.8%)	7 (22.6%)	
**Presence of signs during suction in the initial speech -language evaluation**. n (%)			
Body organization	19 (100.0%)	27 (87.1%)	0.103
Intraoral pressure	18 (94.7%)	17 (54.8%)	0.003**
Suction rhythmic movement	17 (89.5%)	22 (71.0%)	0.125
Respiratory discomfort	0 (0.0%)	5 (16.1%)	0.065
**Speech therapy recommendation of the feeding route after the initial speech -language evaluation**. n (%)			
Exclusive orogastric tube	1 (5.3%)	8 (25.8%)	0.006**
Exclusive nasogastric tube	0 (0.0%)	1 (3.2%)	
Exclusive oral route	10 (52.6%)	3 (9.7%)	
Mixed (SOG or SNG + VO)	8 (42.1%)	19 (61.3%)	
			
			
**Time between the removal of invasive mechanical ventilation and the initial speech -language evaluation**. in days			
mean (± SD)	30.9 (±15.5)	23.6 (±16.4)	0.280
median (min; max)	28.0 (12; 60)	19.5 (3; 63)	
**Time between removal of noninvasive mechanical ventilation and initial speech -language assessment**. in days			
mean (± SD)	12.5 (±6.4)	26.9 (±16.1)	0.218
median (min; max)	12.5 (8; 17)	23.0 (6; 56)	
**Time between the introduction of enteral diet and the initial speech -language evaluation**. in days			
mean (± SD)	1.1 (±2.7)	2.7 (±6.9)	0.064
median (min; max)	0.0 (0; 10)	1.0 (0; 37)	
**Speech therapy sessions between the initial evaluation and the introduction of oral route** in URV			
mean (± SD)	0.2 (±0.5)	1.3 (±2.2)	0.057
median (min; max)	0.0 (0; 2)	0.0 (0; 10)	
**Speech therapy sessions between the initial evaluation and the removal of the probe (SOG or SNG)**. in URV			
mean (± SD)	3.6 (±2.3)	9.6 (±6.1)	<0.001*
median (min; max)	3.0 (1; 8)	9.0 (2; 29)	
**Time between initial speech -language assessment and discharge from speech therapy**. in days			
mean (± SD)	3.5 (±2.3)	22.4 (±19.2)	<0.001*
median (min; max)	2.0 (1; 7)	15.0 (8; 75)	
**Time between initial speech -language assessment and hospital discharge**. in days			
mean (± SD)	13.1 (±17.0)	32.3 (±26.7)	<0.001*
median (min; max)	8.0 (2; 69)	21.0 (8; 95)	

* Significant difference according to the Mann-Whitney test;

** Significant difference according to Pearson’s chi-square test

**Caption**: NE = enteral nutrition; n: number of participants; SD = standard deviation; min: minimum; max: maximum; SNAPPE II = *Score for Neonatal Acute Physiology. Perinatal Extension. Version II*; URV = unit of income value; SOG = orogastric tube; NGS = nasogastric tube; NPP = peripheral parenteral nutrition; VO = oral route

- Analysis of risk factors associated with prolonged speech therapy:

Table 4 shows the multiple logistic regression analysis to jointly evaluate the association of the characteristics of interest that may influence what was considered the negative outcome (i.e., prolonged speech therapy - more than seven days between the initial evaluation and the speech therapy discharge). The results indicate that the diagnosis of GTQ, the use of invasive mechanical ventilation and the absence of adequate intraoral pressure during the first assessment of sucking were risk factors for a negative outcome. Participants with QTQ were 3.477 times more likely to have prolonged speech-language therapy than participants without this diagnosis. Participants who used invasive mechanical ventilation (GTQ and CG) were 1.704 times more likely to have prolonged speech-language therapy than participants who did not use this ventilation method. Finally, participants who presented adequate intraoral pressure during the first assessment of sucking had a 33% lower chance of prolonged speech therapy than participants who did not show the adequacy of this signal. In summary, the diagnosis of GTQ and the use of invasive mechanical ventilation were risk factors for prolonged speech-language therapy and the presence of adequate intraoral pressure during the first assessment of sucking was identified as a protective factor.

**Table 4:** Multiple logistic regression model after the selection method to explain the negative outcome of the participants - prolonged speech therapy treatment (more than 7 days between the initial evaluation and the speech therapy discharge)

**Risk factors**	**OR**	**IC (95%)**		**p value**
		Lower	Superior	
Gestational age at birth	0.98	0.93	1.03	0.346
Diagnosis of gastroschisis	3.477	0.663	18.247	*<0.001* *
Total length of hospital stay	1.00	0.99	1.00	0.472
Use of invasive mechanical ventilation	1.704	1.337	2.171	0.001*
Use of non -invasive mechanical ventilation	4.190	1.246	14.089	0.786
Presence of comorbidities and/or surgical complications of gastroschisis	3.516	0.950	13.012	0.829
Presence of intestinal atresia	0.578	0.450	0.742	0.458
Presence of sepsis	2.708	0.728	10.073	0.602
Time of use of peripheral parenteral nutrition (PPN)	1.05	0.89	1.23	0.596
Time between birth and introduction of enteral diet	0.99	0.96	1.02	0.628
Days of life in the initial speech -language assessment	1.00	1.00	1.00	0.306
Presence of body organization during suction in the initial speech -language assessment	0.196	0.056	0.691	0.669
Presence of intraoral pressure during suction in the initial speech -language evaluation	0.670	0.080	0.870	0.008*
Presence of rhythmic suction movement during suction in the initial speech -language evaluation	0.288	0.055	1.509	0.990
Presence of respiratory distress during suction in the initial speech -language evaluation	0.578	0.450	0.742	0.436
Time between removal of invasive mechanical ventilation and initial speech -language assessment	1.00	0.92	1.08	0.986
Time between the introduction of enteral diet and the initial speech -language assessment	1.53	0.31	7.50	0.600
Speech therapy sessions between the initial evaluation and the introduction of oral route in URV	1.62	0.57	4.59	0.365
Speech therapy sessions between the initial evaluation and the removal of the probe (SOG or SNG). in URV	0.57	0.20	1.64	0.300
Time between initial speech -language assessment and discharge	2.36	0.64	8.71	0.196
Time between initial speech -language assessment and hospital discharge	0.82	0.53	1.29	0.391

* Statistically significant result. According to multiple logistic regression. *Stepwise backward* selection method

**Caption**: OR: *odds ratio*; 95% CI: 95% confidence intervals

## DISCUSSION

In general, the results of the present study indicated that the feeding skills of newborns with QTG are directly related to the severity of the condition (gastrointestinal tract complications), requiring longer hospitalization, use of invasive mechanical ventilation, prolonged use of alternative feeding and requiring more speech therapy to start oral feeding. In our institution, patients are discharged only after being released from speech therapy, i.e., after the removal of the alternative feeding route. The multivariate analysis indicated that the factors related to the negative speech-language outcome, i.e., the need for speech-language intervention for more than 7 days for the introduction of the oral feeding route, were the diagnosis of GTQ, the use of invasive mechanical ventilation and the absence of adequate intraoral pressure in the first speech-language evaluation.

Considering the group of premature infants with GTQ, it was possible to observe the occurrence of sepsis in more than half of the sample. According to the literature, the association between prematurity and sepsis is frequent and is one of the main causes of morbidity and mortality in NB ^(20)^. In general, sepsis in premature infants is due to several factors, such as deficiency in the immune response, making these patients more susceptible to infections. In premature patients with GTQ, there are additional factors that increase the vulnerability of the immune systems of these patients, such as visceral exposure to the external environment, need for prolonged PPN (in our sample. the average PPN time was 60 days) and surgical approaches with surgical wound infection ^(21)^. The occurrence of sepsis impairs clinical stability, prolongs the need for mechanical ventilation and delays the onset of oral feeding. Another factor that stood out in the group with GTQ was the occurrence of cholestasis in 38% of the study sample. According to the literature, cholestasis is associated with the use of prolonged PPN (>3 weeks) ^(21,22)^.

According to the internal protocols of the institution where the study was conducted, the feeding skills of premature NB can only be evaluated when infants reach 33-34 full weeks of gestational age and has clinical stability compatible with the evaluation (e.g., weaning from mechanical ventilation, invasive and noninvasive, cardiorespiratory stability, etc). However, the literature has pointed out the benefits of early assessment of oral feeding readiness of preterm NBs, establishing some criteria, such as gain in control of autonomous functions (heart and respiratory rate and rhythm) , clinical stability and specific feeding behaviors ^(23,24)^. In the present study, both groups of infants had approximately 40 weeks of gestational age at the time of the first speech-language assessment. According to the literature, several factors can influence the ideal time to perform the assessment of oral feeding readiness in each child, and the skills required for oral feeding are usually present after 32 full weeks of gestational age ^(23)^. There is evidence that preterm infants have physiological signs of stability and feeding-related behaviors-demand reflex, strong sucking reflex and hand-mouth behaviors-at 32 weeks of gestational age ^(23)^. Birth weight has been identified as a better factor for determining nutritional status and future growth patterns than gestational age. In addition, birth weight has been considered a biological condition capable of influencing the cognitive and motor development of children ^(24,25)^. Considering these points, the criteria for initiating oral feeding stimulation should be individualized and based on the capabilities and needs of each NB. This factor should be considered in future studies, assessing the impact of early oral feeding stimulation in premature infants with QTQ on clinical and hospital discharge parameters.

The results of the present study indicated that most NBs with GTQ passed the parameters evaluated for NNS at the time of the first speech-language assessment and approximately 30% failed in the assessment of NS. Due to the NICU criteria for requesting the assessment of feeding skills of NBs, it is not possible to determine whether these NBs with GTQ could start feeding stimulation earlier. Several studies that investigated the use of NNS as a form of stimulation for the onset of oral feeding, indicate that NBs subjected to this form of stimulation have greater feeding efficiency, early onset of oral feeding and a better maturation of the feeding skills ^(26,27)^. The literature also indicates that the stimulation of NNSs during alternative feeding accelerates the organization and efficiency of sucking patterns in premature NBs (24,25). Additionally, according to the literature, NBs who are less mature at birth and/or have a higher degree of morbidity tend to take longer to start oral feeding and achieve exclusive oral feeding ^(26)^. In the present study, NBs with GTQ required twice as many speech-language interventions to initiate the transition to the oral feeding and this number was even higher when considering the GTQ group who were submitted to staged surgery.

It is known that the use of ventilatory support and the use of alternative feeding methods delay the stimulation of the oral functions and oral feeding attempts ^(27,28)^. The results of the present study indicated that NBs with GTQ made more use of invasive mechanical ventilation than NBs without comorbidities. The associations between the use of orotracheal intubation and feeding difficulties are related to the impact of the presence of the tube in the oral, pharyngeal and laryngeal cavities, considering that the swallowing reflex is triggered by mechanoreceptors located in the mucosa that lines the pharynx and larynx. These receptors end up undergoing modifications due to the prolonged permanence of the orotracheal tube in these cavities ^(29)^. In addition, the nasogastric feeding tube may interfere with ventilatory support for breathing, as well as with the sucking-swallowing-breathing coordination. Orogastric tubes have also been identified as a negative factor for the initiation of oral feeding because they have the potential to alter the oral reflexes necessary for this function ^(30)^. As observed in the literature, the duration of the feeding tube in the oral cavity, associated with the lack of oral stimulation, may result in a delay in the progression to oral feeding ^(28)^.

Finally, our study had some limitations. First, the gathered data were derived from a single institution and, therefore, may reflect local characteristics of the patients and unique attributes of the procedures adopted in the NICU, such as the time to start oral feeding stimulation. Considering the great variability of practices involving feeding in NICUs of different institutions, the findings presented in our study cannot be generalized. However, the sample consisted of newborns with GTQ who underwent surgical correction and were referred to a feeding evaluation. Thus, we believe that our results represent this patient population. As a second point, the present study is retrospective, therefore, a few data, such as the difference in weight gain before and after oral feeding stimulation, were not recovered. These data should be considered in future studies since weight gain is usually associated with shorter stays in the NICU and lower hospital costs. In addition, these data may help to better understand the clinical markers associated with the feeding skills of NBs with GTQ.

## CONCLUSION

The feeding skills of preterm infants with QTG are related to the severity of the condition (gastrointestinal tract complications), requiring longer hospitalization, use of invasive mechanical ventilation, prolonged use of alternative feeding methods and requiring more speech therapy to begin the treatment of oral feeding.
